# Editorial: Gene and Environment Interactions in Neurodevelopmental Disorders

**DOI:** 10.3389/fnbeh.2022.893662

**Published:** 2022-03-31

**Authors:** Patrícia Pelufo Silveira, Lorenzo More, Carmem Gottfried

**Affiliations:** ^1^Ludmer Centre for Neuroinformatics and Mental Health, Douglas Mental Health University Institute & Department of Psychiatry, McGill University, Montreal, QC, Canada; ^2^School of Pharmacy and Biomedical Sciences, University of Central Lancashire, Preston, United Kingdom; ^3^Departamento de Bioquímica, Instituto de Ciências Básicas da Saúde, Universidade Federal Do Rio Grande Do Sul, Porto Alegre, Brazil

**Keywords:** gene environment interaction, development, early adversity, prenatal stress, genetics

The knowledge that both the genetic patrimony and lifetime environmental exposures define disease risk is well-accepted. However, the so-called Gene-Environment effects—when the consequences of an environmental exposure vary according to the genetic background—are less understood (Kloke et al., 2013). This is especially true in early life when individuals undergo a series of dynamic and tightly linked developmental processes (Miguel et al., 2019a). The brain and the periphery reorganize during specific developmental time periods to adapt to changes in the environment the subjects are being raised. These are known as “critical periods” (Hensch, 2004).

There has been an increased interest in unraveling how certain types of exposure occurring during these critical periods affect developmental trajectories. Characterizing relevant genes and proteins involved and their precise timing of action in relationship with the type and severity of the environmental exposure is critically needed and was the main aim of this Research Topic. The articles enclosed in this Research Topic represent important advances in the understanding of these relationships.

Focusing on main genetic effects, Zhongling et al. reported a case of Joubert syndrome associated with a new mutation in IFT74, a gene responsible for regulating cilia composition. Wang Y. et al. identified increased allele frequencies of single nucleotide polymorphisms (SNPs) from the Interleukin-23 (IL-23) gene in children with cerebral palsy compared to healthy controls. The perspective from Malatesta et al. focuses on the environmental stimulus, proposing the existence of a critical period during which caregiver's postural and motor lateral biases influence offspring hemispheric lateralization.

During gestation, fetal environment is defined by the maternal metabolic milieu, which influences fetal development. Wang H. et al. investigated the effects of high maternal estradiol on proliferation and differentiation of fetal hypothalamic neural stem/progenitor cells (NSC/NPCs) in mice and identified critical effects on neurogenesis related genes. Sandoval et al. explored another maternal internal state during pregnancy: immune activation, which usually results from infections. Though maternal immune activation induced behavioral alterations compatible with autism spectrum disorders and schizophrenia in the offspring, this was not exacerbated by the loss of vesicular zinc, another known risk factor for neurodevelopmental disorders.

Szekely et al. compiled five candidate polymorphisms (one per gene: DAT1, DRD4, DRD2, COMT, BDNF) in a multilocus score, to explore their interaction with prenatal adversity and postnatal parenting behavior on the development of attentional competence skills in 18- and 24-months children. The same candidate polymorphisms representing COMT and BDNF were used in the study from Cao-Lei et al., investigating the effects of prenatal maternal stress (defined by exposure to a natural disaster during pregnancy) on hippocampal volumes at 11–12 years of age. The SNP located on COMT gene moderated the effect of maternal distress on hippocampal volumes, suggesting that gene-environment interactions have long-term effects on brain neuroanatomical features.

de Mendonça Filho et al. used a novel approach to genomic profiling (Silveira et al., 2017; Hari Dass et al., 2019; Miguel et al., 2019b), representing variations in a prefrontal cortex-specific BDNF gene co-expression network in children, and show that this biological mechanism moderates the effect of prenatal adversity on cognitive developmental trajectories between 6 and 36 months. Intriguingly, epigenetics-related components of the BDNF gene network moderate the effects of prenatal adversity on gray matter content in cortical regions later in childhood. The same approach (Silveira et al., 2017; Hari Dass et al., 2019; Miguel et al., 2019b) was employed by Potter-Dickey et al. to investigate if prefrontal, striatal and hippocampal Cannabinoid Receptor 1 (CNR1) gene co-expression networks moderate the effect of parental caregiving quality on infant attachment styles. Finally, Batra et al. demonstrated that the genetic background associated with higher fasting insulin regulates the effects of early adversity on the development of inhibitory control in young children, corroborating the programming effects of insulin on executive functions in response to early adversity (Batra et al., 2021).

The Research Topic compiles evidence that gene-environment interactions influence neurodevelopment, proposing mechanisms by which this moderation occurs (Figure 1). The studies illustrate novel techniques that can uncover biological targets and pathways with important implications for early detection, prevention, and treatment of neurodevelopmental disturbances.

**Figure 1:**
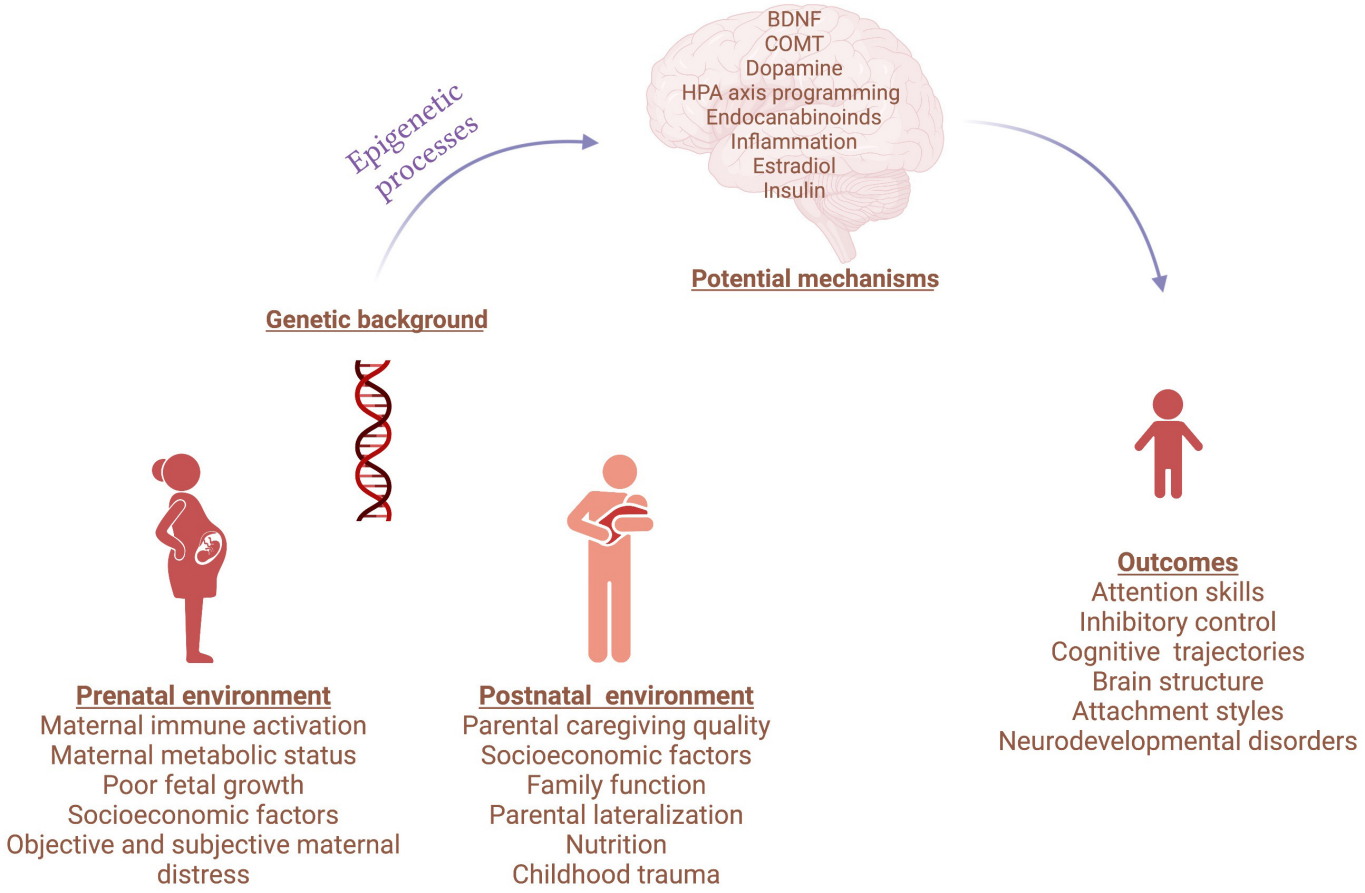
Gene and environment interactions in neurodevelopmental disorders. Created with BioRender.com.

## Author Contributions

All authors wrote and approved the submitted version.

## Conflict of Interest

The authors declare that the research was conducted in the absence of any commercial or financial relationships that could be construed as a potential conflict of interest.

## Publisher's Note

All claims expressed in this article are solely those of the authors and do not necessarily represent those of their affiliated organizations, or those of the publisher, the editors and the reviewers. Any product that may be evaluated in this article, or claim that may be made by its manufacturer, is not guaranteed or endorsed by the publisher.
